# Online Tourism Information and Tourist Behavior: A Structural Equation Modeling Analysis Based on a Self-Administered Survey

**DOI:** 10.3389/fpsyg.2020.00599

**Published:** 2020-04-21

**Authors:** Salman Majeed, Zhimin Zhou, Changbao Lu, Haywantee Ramkissoon

**Affiliations:** ^1^Department of Marketing, College of Management, Shenzhen University, Shenzhen, China; ^2^School of Economics and Management, Fuzhou University, Fuzhou, China; ^3^Derby Business School, College of Business, Law and Social Sciences, Derby, United Kingdom; ^4^Monash Business School, Department of Marketing, Monash University, Melbourne, VIC, Australia; ^5^School of Business and Economics, Faculty of Biosciences, Fisheries and Economics, The Arctic University of Norway, Tromsø, Norway; ^6^College of Business and Economics, University of Johannesburg, Johannesburg, South Africa

**Keywords:** online tourism information, tourist perceptions, satisfaction, tourist behavior, electronic word-of-mouth, destination marketing

## Abstract

This study presents the interacting phenomena of perceptions of tourist destination online content (TDOC) and tourists’ behavioral intentions with a mediating role of tourists’ satisfaction, which is as yet under-explored in hospitality and tourism research. A model based on three main constructs, namely TDOC (with sub-constructs of online information quality and user-friendly accessibility), satisfaction, and tourists’ behavioral intentions [with sub-constructs of intentions to visit a tourist destination and electronic word-of-mouth (eWOM)], is presented to determine the growth of tourism business with the internet. Data were collected via a questionnaire-based survey from 413 tourists staying at hotels in Lahore city in Pakistan. Partial least square structural equation modeling was used to statistically analyze the gathered data. The findings indicate that tourists’ perceptions of TDOC directly influence their behavioral intentions, while tourists’ satisfaction exerts a mediating influence between tourists’ perceptions of TDOC and their behavioral intentions. Taking advantage of an economical and widespread online environment, destination marketing organizations could attract more tourists by fostering confidence in TDOC and positive eWOM to remain competitive in the long run. Important theoretical and practical implications are discussed.

## Introduction

Tourists start searching for online information about the best tourist destination and hotels before planning their actual traveling schedule (Nunkoo and Ramkissoon, 2013; Ramkissoon, 2018a, b). The internet has remained a predominant mode of information collection for leisure planning. The possession of information communication technology (ICT) devices, such as laptops, tablets, iPads, and smartphones, among tourists has fueled the use of the internet in tourism planning, promotion, and consumption across the globe (Parra-López et al., 2011). Due to the unprecedented growth of e-commerce in the service industry, tourist destination information might be available on different e-platforms, such as social media, search engines, websites, and e-blogs. This has contributed significantly to the promotion of tourism and hospitality business worldwide. The latest ICT and the internet have enabled tourists to quickly search, interact, compare, and make decisions to purchase online tourism and hospitality deals (Mills and Law, 2004; Law et al., 2018).

Tourists’ satisfaction with tourist destination online content (TDOC), such as the quality of online information on tourism deals and its accessibility on the internet, may help shape their positive emotions, such as happiness, brand loyalty, and intentions to visit a tourist destination and to spread positive electronic word-of-mouth (eWOM) (Saleem et al., 2018). Scholars define eWOM communication as customers’ positive or negative statements on the internet about the product, service, people, and institutions (Jalilvand et al., 2012). Tourism offerings and bonus deals promoted through a destination’s online platform, such as social media and websites, may determine tourists’ behavioral intentions to visit destinations. Some scholars have examined marketing strategies aimed at promoting a destination’s online tourism and hospitality business (Fesenmaier and Xiang, 2014; Pino et al., 2018). A destination’s tourism promotion via technology corridors, e.g., mobiles, tablets, computers, the internet, etc., has also been analyzed (Kim et al., 2014; Dewnarain et al., 2019; Huang et al., 2019). However, the role of TDOC in determining tourists’ behavioral intentions remains under-explored.

In the context of purchasing a product or service from the virtual market, the quality of the information in terms of accuracy, quality of graphical images, completeness, and accessibility is considered in online information search (Parra-López et al., 2011; Ramkissoon and Uysal, 2011). This is further fueled by the unprecedented growth of social media, e-blogs, websites, and search engines where customers can enter into interactive communications with the service providers and read comments from existing consumers regarding the product or service. Such online communications may impact information-seeking consumers’ perceptions, satisfaction, happiness, and behaviors, e.g., eWOM, travel intentions, etc. (Parra-López et al., 2011; Sun et al., 2016; Saleem et al., 2018).

Tourism falls into the category of IHIP (intangible, heterogeneous, inseparable, perishable) services and is considered a product that cannot be consumed before its experience (Moeller, 2010). Considering the unknown quality of tourism and the fact that the internet has majorly influenced consumers’ information search and purchase behaviors, there is a need to explore how tourists perceive and respond to TDOC. Some scholars have analyzed tourists’ destination choice behaviors based on perceptions and behavior models (Chen et al., 2014; Chung et al., 2015; Majeed et al., 2018). However, within the diversified realms of online marketing and advertising, researchers are yet to empirically examine the underlying phenomenon that pushes tourists’ favorable responses toward TDOC.

Since consumers’ online search behaviors are helpful for predicting their buying intentions from virtual markets, smart business technologies focus on gathering information on users’ online browsing history to influence their purchase decisions, satisfaction, happiness, and contagion behaviors (Mills and Law, 2004; Lee et al., 2018). Tourist destination organizations attempt to grab the attention of online information-seeking tourists through their presence at different online media platforms in an attempt to offer accurate and reliable information, which might encourage existing consumers to comment online in few clicks so that tourists may feel positive about a tourist destination through their online-browsing experience (Sabiote-Ortiz et al., 2016; Sun et al., 2016; Aydin, 2019).

Positive online browsing experience may lead to individual-level satisfaction, which is considered an antecedent to pleasant moods and behavioral intentions, such as favorable intentions to visit a tourist destination and to spread positive eWOM (Jalilvand et al., 2012; Ting et al., 2013). Tourists’ satisfaction with TDOC, such as online information quality and user-friendly accessibility, may significantly influence their travel behavioral intentions. The emergence of virtual markets for online shopping, however, often creates doubt in users about the security and reliability of online platforms. Threats in the online business environment, such as the risk of losing personal information and payment identification information, may dissuade customers from purchasing online. This may further determine tourists’ positive and negative behaviors toward online purchasing of tourism and hospitality services (Kim et al., 2008; Lin et al., 2009). The association of human behavior with satisfaction is oft quoted in the literature on psychology (Weber and Johnson, 2008; Ramkissoon and Mavondo, 2015). The tourism literature also explores the relationship between satisfaction and tourists’ behavioral intentions (e.g., Ramkissoon et al., 2012, 2013a,b; Sabiote-Ortiz et al., 2016). However, the mediating impact of satisfaction on the relationship between tourists’ perceptions of TDOC and their behavioral intentions is yet to be analyzed in the online stream of tourism business.

This study is highly focused on examining whether tourists’ perceptions of TDOC, including of online information quality and user-friendly accessibility, can impact their behavioral intentions, including intentions to visit tourist destinations and distribute eWOM, and their satisfaction. We also attempt to analyze the mediating role of satisfaction in the relationship between tourists’ perceptions of TDOC and their behavioral intentions. Our empirical findings from Lahore, the capital city of Punjab province in Pakistan, which is an emerging tourism market, provide a different perspective to extend the theory and practice of TDOC. Our findings contribute to the tourism and hospitality literature in investigating the associations between dimensions of TDOC and tourists’ behavioral intentions and the mediating impact of tourists’ satisfaction in a single integrative model. Our findings bridge the theoretical gaps in the online and wireless business frontiers of tourism and hospitality. This study provides roadmaps to the marketers of tourist destinations to attract tourists by offering a satisfying online browsing experience, which may assist in attracting more tourists and generating positive eWOM. The stakeholders of tourism and hospitality may boost their business in the light of findings uncovered under the empirical lens of this study. Thus, this study provides a useful reference for online tourism marketing and destination management organizations with its robust theoretical and practical implications.

The remainder of the article is as follows: first, a literature review is presented to provide insights into the concepts of tourists’ perceptions of TDOC, satisfaction, and behavioral intentions, underpinned by theory. Hypotheses are presented that are supported by relevant extant literature. The methodology and research settings are then detailed, followed by the discussion of findings. Limitations and avenues for future research are further discussed in the concluding section.

## Literature Review

With the introduction of virtual markets, the importance of computers, the internet, web technologies, and electronic marketing have become prominent in lubricating the buying and selling functions of goods and services. Virtual markets have increased the profit-earning capacity of firms with their cost-effective philosophy under the influence of the pervasive roles of the internet and computer–human interaction (Zhang et al., 2011). Online tourism marketing has attracted tourists from almost every corner of the world (Coenders et al., 2016; Sabiote-Ortiz et al., 2016). The tourism industry encapsulates many service industries in its breadth and depth, such as food and beverages, travel, and hospitality, with a variety of niches, e.g., wellness tourism, medical tourism, spa tourism, etc. (Majeed and Lu, 2017; Majeed et al., 2017a). The tourism industry is experiencing a boom in its service operations due to tourists’ demands for their overall health and well-being (Sabiote-Ortiz et al., 2016; Majeed et al., 2017a, 2018, 2019a; Aydin, 2019). In this rapidly evolving era of tourism, the internet, and ICT, destinations are more easily explored than before. Scholars note that the combined influence of the internet and ICT makes a major contribution to the promotion of tourism and hospitality businesses across the globe (Mills and Law, 2004; Parra-López et al., 2011; Pino et al., 2018).

Tourist destination online content is a multi-textured concept that is deeply linked to the availability of online information about tourism. Scholars note that tourists feel comfortable with the easy accessibility of online information about their potential tourist destinations (Saleem et al., 2018). Tourists’ pre-purchase travel decisions depend on the quality of online tourism information and its easy availability on different online platforms (e.g., Parra-López et al., 2011; Ting et al., 2013; Huang et al., 2019). Thus, the concept of TDOC can arguably be stated in terms of online information quality and user-friendly accessibility of tourism information in the online environment.

Tourists’ behavioral responses toward a destination are shaped by their perceptual filters (Majeed et al., 2019b). TDOC passes through tourists’ cognitive evaluation, such as knowledge and belief, and affective appraisals, such as feelings, brand purchasing, and actual traveling behaviors (Baloglu and Brinberg, 1997; Pike and Ryan, 2004; Nicoletta and Servidio, 2012; Xue et al., 2020). Drawing on TDOC, tourists’ perceptions are focused on in this study as the best lens through which to explore tourists’ behavioral responses to online tourism content.

## Hypothesis Development

### TDOC and Satisfaction

The relationship between an individual’s perceptions and behaviors has been explored in marketing and psychology literature (Xue et al., 2020). Expectation confirmation theory (ECT) notes that individuals’ expectations, perceptions, and satisfaction are interlinked phenomena and are important antecedents to individuals’ behavioral responses (Oliver, 1997). Individuals’ unique perceptual filters determine their positive or negative experience of expected service and satisfaction level (Oliver, 1997). ECT notes that individuals may be satisfied if their perceptions of a product or service exceed their expectations. Likewise, individuals may be dissatisfied if their perceptions of a product or service are not met according to their expectations (Oliver, 1997; Ye et al., 2019). From the perspective of tourism and hospitality, the interplay of tourists’ expectations and perceptions determines their satisfaction with tourism services (Majeed and Lu, 2017; Majeed et al., 2018; Ye et al., 2019).

Tourists’ satisfaction depends on the information quality of TDOC (Ting et al., 2013). However, the evaluation of tourists’ satisfaction is a difficult phenomenon confronting the tourism business because of tourists’ complex perceptual filters on experiencing tourism quality (Majeed et al., 2018). Destination marketing practitioners struggle to explore how to bring satisfaction to tourists through online tourism promotion (Fernandes and Fernandes, 2018).

The high quality of tourism service in terms of reliability and completeness of information may deliver satisfaction to online information-seeking tourists. This study further draws on the theory of planned behavior (Ajzen, 1991), which posits that individuals’ perceptions largely determine their satisfaction.

Tourists search for the best tourism and hospitality offerings at different online platforms before making decisions to travel to their desired tourist destination (Nunkoo and Ramkissoon, 2013; Coenders et al., 2016). Tourists purchase tourism deals from the virtual market when online information is perceived to be accurate and reliable. The perceived quality of TDOC may attract or discourage tourists from transacting with the service provider in the virtual market. Tourists might feel at risk of being cheated or suffering losses when purchasing online tourism packages if the information is not reliable. Tourists’ doubts about the authenticity of TDOC may give rise to dissatisfaction with the use of online platforms (Kim et al., 2008; Jalilvand et al., 2012; Sun et al., 2016).

The stakeholders of the tourism industry pump up their profit balloons by capitalizing on the unprecedented growth of online tourism marketing. The internet allows marketing practitioners and tourists to interact with each other in real time. The marketing efforts of tourist destinations will be more productive when reliable, complete, and well-grounded TDOC is available, which contributes to tourists’ satisfaction (Fesenmaier and Xiang, 2014).

Destination Marketing Organizations attract tourists with different tactics (Hristov and Ramkissoon, 2016; Ramkissoon and Hristov, 2018), such as the marketing of low-price tourism packages. Despite the availability of such information on the internet, the quality of online service also contributes to the survival of a business. The roots of service quality are grounded in the theoretical understanding of determining tourists’ satisfaction with the quality of online tourism service, which is different from analyzing the attributes of service quality during tourists’ actual interaction with the tourist destinations (Bastida and Huan, 2014).

The concept of online service quality is linked to higher customer satisfaction (Saleem et al., 2018). The importance of the internet marketing in fueling e-tourism business and, via a satisfactory browsing experience, attracting tourists to avail of online tourism deals has been acknowledged and, thus, tourist destinations are increasingly presenting information on their tourism products on different online platforms (Wang and Pizam, 2011; Cao and Yang, 2016). Tourist destinations may become highly competitive markets with good profit margins if tourists are satisfied with the perceptions of TDOC because individuals’ satisfaction is reflective of their perceptions.

Based on the above, the following hypothesis is proposed.

Hypothesis H1: Perceptions of TDOC will have a significantly positive impact on tourists’ satisfaction.

### TDOC and Tourists’ Behavioral Intentions

Tourists’ compatibility with TDOC, which is determined by attitudes, may affect their favorable intentions to visit tourist destinations (Duarte and Amaro, 2015). Perceiving reliable, accurate, and easily available TDOC may reduce perceived risk and fuel positive intentions to visit tourist destinations (Kuo et al., 2015). Scholars recognize that positive perceptions of TDOC may attract tourists and give rise to positive intentions to visit tourist destinations (Woodside et al., 2011).

Scholars have noted that online marketing helps to promote e-tourism business at different online platforms and attempts to attract tourists to online tourism deals with reliable tourism content to boost tourists’ confidence so that they can develop positive intentions to visit tourist destinations (Wang and Pizam, 2011; Cao and Yang, 2016; Saleem et al., 2018).

Since tourism is being promoted on different platforms, such as social media, website, e-blogs, and search engines, TDOC is considered appropriate and usable only if easily available to tourists during their initial online browsing of tourism information. User-friendly online information is reflected in terms of the good quality of images (if any), appropriate font size, logical text links and navigation, completeness and clarity of information, payment guarantee, security and privacy policies, and authentic contact details of the service provider (Wang and Emurian, 2005; Seckler et al., 2015). User-friendly accessibility of TDOC may give rise to positive perceptions, high trust and satisfaction levels, and loyalty of tourists to a host tourist destination, causing them to give positive recommendations. Tourism marketing professionals promote TDOC with unique tourism products and services. Thus, tourists’ positive intentions to visit tourist destinations may be associated with the concepts of easy accessibility, usability, and appropriateness of TDOC.

Scholars note that TDOC may give rise to positive or negative eWOM among online information-seeking tourists (Jalilvand et al., 2012; Sun et al., 2016). The importance of internet marketing in fueling e-tourism business and attracting tourists with favorable purchasing intentions alongside positive eWOM has been acknowledged (Wang and Pizam, 2011; Cao and Yang, 2016). Tourists’ doubts on the authenticity of TDOC may generate dissatisfaction and negative eWOM about the destination (Kim et al., 2008; Jalilvand et al., 2012; Sun et al., 2016). Behavioral intentions in this study are reflected in terms of tourists’ intentions to visit a tourist destination and eWOM, and the following hypothesis is proposed.

Hypothesis H2: Perceptions of TDOC will have a significantly positive impact on tourists’ behavioral intentions.

### Satisfaction and Tourists’ Behavioral Intentions

A plethora of research has examined tourists’ purchase behaviors for tourism products and services, intentions to visit tourist destinations, and information-sharing behaviors regarding their tourism experience, but tourists’ information search behaviors and their concerns about the quality and accessibility of TDOC are as yet under-explored. Moreover, within the diversified realms of online marketing and advertising, scholars are yet to explore the underlying phenomenon determining tourists’ favorable responses toward TDOC.

Feelings of being cheated, loss of important information, or insecurity while browsing online content are counted as perceived online browsing risk (Alcántara-Pilar et al., 2015). Consumers’ perceptions of the credibility of TDOC and satisfaction primarily depend on their initial feelings about perceived online browsing risk while accessing online information for the first time, which is linked to generating satisfaction or dissatisfaction among tourists (Lin et al., 2009). Consumers’ behaviors in the business environment, such as purchasing a product or service and sharing comments with others, are largely determined by their satisfaction versus the level of perceived risk in business dealings (Vainikka, 2013).

Tourism encapsulates higher risk and lower tourist satisfaction due to its IHIP (intangible, heterogeneous, inseparable, and perishable) dimensions. Consumers of a service may become more dissatisfied in dealing with the service providers as compared to the consumers of a tangible product (Murray and Schlacter, 1990; Albayrak et al., 2010). This situation arises because of the virtual nature of buying and selling products and services, with no physical contact with the service provider (Wang and Emurian, 2005). Since the internet and ICT have pervasively infiltrated the tourism and hospitality segments, tourists’ dissatisfaction is also at its maximum in the recent era of e-commerce.

Tourists browse online tourism information and prefer to compare and purchase the best online tourism packages before in traveling to tourist destinations with their family and friends (Yang and Nair, 2014; Ramkissoon, 2018a). Perceptions of TDOC may influence tourists’ satisfaction and behavioral intentions, such as purchasing of tourism deals, physically visiting tourist destinations, and spreading word-of-mouth (Ting et al., 2013). Low perceived browsing risk in TDOC may help to deliver satisfying browsing experience to tourists alongside positively influencing their behavioral intentions (Loewenstein et al., 2001; Yang and Nair, 2014).

Oliver (2010) argues that individual-level satisfaction is important for consumers’ positive behaviors. Individuals’ satisfaction is the outcome of their perceptions, which may generate positive or negative feelings (Oliver, 1997, 2010). Some research presents the relationship between tourists’ satisfaction and their behaviors in the different contexts of the tourism industry (Sirgy and Su, 2000; Majeed et al., 2018, 2019b). The majority of the research on tourist satisfaction reflects the conventional perspectives without considering how satisfaction with tourism offerings mediates tourists’ perceptions and behaviors in the rapidly development environment of the internet and ICT.

Some evidence shows that customers’ satisfaction with TDOC positively affects their favorable intentions of purchasing the advertised products and services (Jalilvand et al., 2012; Ting et al., 2013). Thus, tourists’ perceptions of TDOC and resultant satisfaction play instrumental roles in their developing positive behavioral intentions (Fernandes and Fernandes, 2018). Tourists’ favorable decisions to buy online tourism services depend on the information quality and user-friendly accessibility features of TDOC, which may further influence tourists’ positive intentions to visit a tourist destination and eWOM. Tourists’ positive or negative feelings about traveling to a destination are developed during their first exposure to TDOC (Chung et al., 2015). Negative online browsing experience may dissatisfy tourists, which will act as a stimulus to doubts developing in the minds of tourists about their online purchasing decision-making (Jalilvand et al., 2012). The majority of dissatisfied tourists spread negative eWOM about their tourism experience and may choose another destination for their future leisure traveling. Tourist destinations attempt to satisfy tourists with their tourism offerings and to develop tourists’ intentions to visit in parallel with spreading positive eWOM, so that they may remain competitive in the long run (Saleem et al., 2018). Tourists are increasingly interested in gathering online information before making traveling decisions, and experienced tourists’ satisfactory feedback may have a contagion effect to attract new tourists to TDOC and shape other tourists’ behavioral intentions. Tourists’ eWOM has a strong contagion effect on attracting other tourists to or keeping them away from tourist destinations (Andreassen and Streukens, 2009; Fernandes and Fernandes, 2018; Aydin, 2019). Thus, tourism marketing professionals consider tourists’ positive eWOM to be as important as tourists’ satisfaction and favorable intentions to visit the tourist destination. This is because eWOM is considered by other tourists as unbiased and is a faster, more perceptible way of promoting a destination’s image; it might be difficult to promote the destination’s image in such a rapid way and to satisfy tourists with traditional marketing and word-of-mouth stimuli (Hennig-Thurau et al., 2004; Jalilvand and Samiei, 2012; Bilgihan et al., 2016).

Some scholars have presented tourist decision-making models of destination choice in the online environment (Chen et al., 2014; Chung et al., 2015). The roles of tourists’ perceptions and satisfaction in determining their intentions to visit tourist destinations have also been examined (Lam and Hsu, 2006; Tan and Wu, 2016; Majeed et al., 2018). Considering the unprecedented growth of the internet and ICT in the tourism industry and tourists’ preferences of browsing online information before planning their traveling schedules, there is a need to explore the interwoven connections between tourists’ satisfaction with TDOC and their behavioral intentions. Based on the above, the following is proposed.

Hypothesis 3: Satisfaction will have a significantly positive impact on tourists’ behavioral intentions.

Considering the instrumental association of TDOC, satisfaction, and tourists’ behavioral intentions discussed above, the following hypothesis is also proposed.

Hypothesis 4: Satisfaction will mediate the relationship between tourists’ perceptions of TDOC and their behavioral intentions.

**FIGURE 1:**
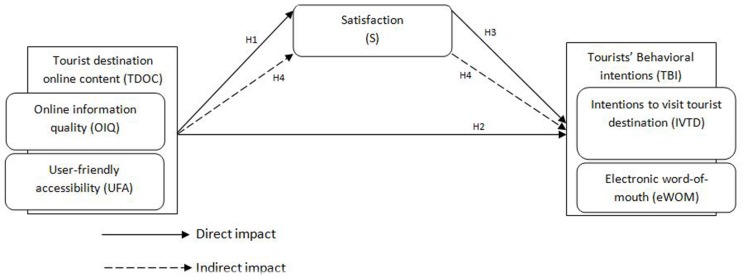
The study theoretical framework.

A theoretical framework is proposed in this study based on the above theoretical underpinnings and hypotheses (see Figure 1).

### Research Model Equations

In this study, TDOC is composed of online information quality (OIQ) and user-friendly accessibility (UFA), while tourists’ behavioral intentions (TBI) is composed of intentions to visit a tourist destination (IVTD) and electronic word-of-mouth (eWOM). The following equations further elaborate the interwoven connections of TDOC, satisfaction (S), and TBI detailed above. We use acronyms of the constructs and sub-constructs of the variable of this study to develop the equations and to explain the interacting phenomena of online tourism and tourists’ behaviors.

For the relationship between OIQ and IVTD mediated by satisfaction:

(1) $$S=a_{0}+a_{1}\mathrm{OIQ}+e_{M}$$

(2) $$\mathrm{IVTD}=b_{0}+b_{1}S+b_{2}\mathrm{OIQ}+e_{Y}$$

Substituting for “S” in Eq. (2):

$$\mathrm{IVTD}=b_{0}+b_{1}(a_{0}+a_{1}\mathrm{OIQ}+e_{M})+b_{2}\mathrm{OIQ}+e_{Y}$$

Thus,

$$\mathrm{IVTD}=(b_{0}+a_{0}b_{1})+(b_{2}+a_{1}b_{1})\mathrm{OIQ}+e_{Y}+b_{1}e_{M}$$

where b_2_ = direct effect, a_1_b_1_ = indirect effect, and b_0_, a_0_, e_Y_, and e_M_ are simple residuals.

For the relationship between OIQ and eWOM mediated by satisfaction:

(3) $$S=a_{0}+a_{1}\mathrm{OIQ}+e_{M}$$

(4) $$\mathrm{eWOM}=b_{0}+b_{1}S+b_{2}\mathrm{OIQ}+e_{Y}$$

Substituting for “S” in Eq. (4):

$$\mathrm{eWOM}=b_{0}+b_{1}(a_{0}+a_{1}\mathrm{OIQ}+e_{M})+b_{2}\mathrm{OIQ}+eY$$

Thus,

$$\mathrm{eWOM}=(b_{0}+a_{0}b_{1})+(b_{2}+a_{1}b_{1})\mathrm{OIQ}+e_{Y}+b_{1}e_{M}$$

where b_2_ = direct effect, a_1_b_1_ = indirect effect, and b_0_, a_0_, e_Y_, and e_M_ are simple residuals.

For the relationship between UFA and IVTD mediated by satisfaction:

(5) $$S=a_{0}+a_{1}\mathrm{UFA}+e_{M}$$

(6) $$\mathrm{IVTD}=b_{0}+b_{1}S+b_{2}\mathrm{UFA}+e_{Y}$$

Substituting for “S” in Eq. (6):

$$\mathrm{IVTD}=b_{0}+b_{1}(a_{0}+a_{1}\mathrm{UFA}+e_{M})+b_{2}\mathrm{UFA}+e_{Y}$$

Thus,

$$\mathrm{IVTD}=(b_{0}+a_{0}b_{1})+(b_{2}+a_{1}b_{1})\mathrm{UFA}+e_{Y}+b_{1}e_{M}$$

where b_2_ = direct effect, a_1_b_1_ = indirect effect, and b_0_, a_0_, e_Y_, and e_M_ are simple residuals.

For the relationship between UFA and eWOM mediated by satisfaction:

(7) $$S=a_{0}+a_{1}\mathrm{UFA}+e_{M}$$

(8) $$\mathrm{eWOM}=b_{0}+b_{1}S+b_{2}\mathrm{UFA}+e_{Y}$$

Substituting for “S” in Eq. (8):

$$\mathrm{eWOM}=b_{0}+b_{1}(a_{0}+a_{1}\mathrm{UFA}+e_{M})+b_{2}\mathrm{UFA}+e_{Y}$$

Thus,

$$\mathrm{eWOM}=(b_{0}+a_{0}b_{1})+(b_{2}+a_{1}b_{1})\mathrm{UFA}+e_{Y}+b_{1}e_{M}$$

where b_2_ = direct effect, a_1_b_1_ = indirect effect, and b_0_, a_0_, e_Y_, and e_M_ are simple residuals.

This study presents the direct relationship of the TDOC constructs of OIQ and UFA with IVTD and eWOM alongside the indirect associations under the mediating pressure of satisfaction.

## Methodology

For data collection, samples were drawn, with a judgmental sampling approach, from tourists staying at hotels in Lahore from the second week of January 2019 to the fourth week of May 2019. Hotels were chosen based on initial screening at Booking.com, which is one of the best hotel-booking websites (McCormick, 2019; Unger, 2019). Based on the star rating at Booking.com, there are 8 5-star hotels, 16 4-star hotels, 41 3-star hotels, and 15 2-star hotels in Lahore. Hotels were contacted via emails to get permission and access to tourists for the data collection. The purpose and objectives of this study were briefly mentioned in the emails to hotel authorities. Seventeen hotels, i.e., one 5-star hotel, three 4-star hotels, eight 3-star hotels, and five 2-star hotels, allowed the study to be conducted at their physical locations in Lahore. Data were collected by hotel managers because the researchers of the present study were not allowed to directly contact tourists staying at participating hotels in Lahore. However, the managers of the hotels assured the authors of the present study that they would collect responses while maintaining the confidentiality and anonymity of respondent tourists. This study was conducted in conformity with the recommendations of the ethics committee of Shenzhen University, Shenzhen, China. The study respondents provided their written informed consent in accordance with the Declaration of Helsinki.

A total of 25 items for the questionnaire were adapted from previous studies and adjusted for the development of the survey questionnaire (see Table 1). Appendix 1 presents the study questionnaire, which used seven-point Likert scale, i.e., strongly disagree (1) to strongly agree (7), to measure the study items. A blind translation back-translation method was followed (Soriano and Foxall, 2002) to translate the original English version of the questionnaire into Urdu, which was reviewed by two doctoral students who were Native Urdu speakers and were proficient in English. Later, both English and Urdu versions of the questionnaires were compared by three bilingual professors, who were not familiar with the original questionnaire, to compare the content of the scale items. After reasonable comparison and minor corrections to the Urdu-translated questionnaire, the quality of scale items was guaranteed, and the survey questionnaires were presented to hotel authorities in English with Urdu translation for respondents’ appropriate understanding of scale items. To maximize the content validity of the questionnaire, a pilot study was conducted on 30 conveniently available individuals who had booked online tourism itineraries in their recent past. The wording and expressions of a few items in the questionnaire were adjusted according to respondents’ recommendations.

**TABLE 1:** Questionnaire items.

Construct	No. of items	Source
**Tourist destination online content (TDOC)**		
Online information quality (OIQ)	5	Andreassen and Streukens, 2009; Albayrak et al., 2010; Bragazzi, 2014; Do et al., 2015; Evans, 2015
User-friendly accessibility (UFA)	5	Kirakowski et al., 1998; Do et al., 2015
**Satisfaction (S)**	5	Andreassen and Streukens, 2009; Albayrak et al., 2010; Moore, 2012; Hao et al., 2015
**Tourists’ behavioral intentions (TBI)**		
Intentions to visit tourist destination (IVTD)	5	Baloglu and Brinberg, 1997; Andreassen and Streukens, 2009; Albayrak et al., 2010; Artigas et al., 2015; Evans, 2015
Electronic word-of-mouth (eWOM)	5	Andreassen and Streukens, 2009; Albayrak et al., 2010; Evans, 2015
**Total**	**25**	

**FIGURE 2:**
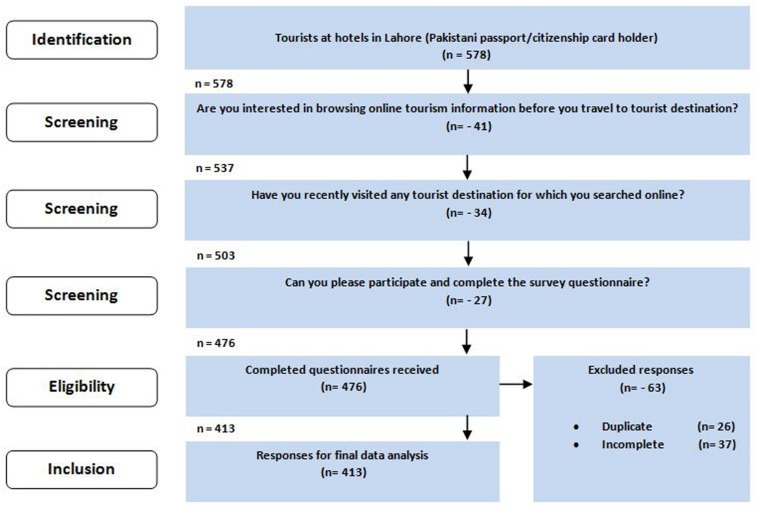
Tourists’ recruitment process at participating hotels.

A total of 600 questionnaires were made available to the participating hotels for final data collection. Several screening questions were made part of the questionnaire for appropriate data gathering, i.e., Are you interested in browsing online tourism information before you travel to tourist destinations? Have you recently visited any tourist destination for which you searched online? Can you please participate and complete the survey questionnaire? (see Figure 2). A total of 476 filled questionnaires were collected from the hotel managers. After scrutinizing them to detect incomplete and duplicate questionnaires, i.e., where the tourist made more than one choice for the same question, a total of 413 questionnaires were retained for final data analysis. The recruitment process of the study respondents alongside the details of finalized responses are shown in Figure 2. The socio-demographic details of tourists (Table 2) show that the majority of the respondents were male (56.90%) and aged between 31 and 50 years (61.50%). Approximately 53.51% of the respondents had completed university. The majority of respondents reported monthly income below USD 2001 (67.07%) and average online browsing time of more than 10 h in a week (68.28%).

**TABLE 2:** Tourist demographics.

Variables	Category	Frequency	Percentage	Cumulative percentage
Gender	Male	235	56.90	56.90
	Female	178	43.10	100
	Total	413	100	
Age (years)	18–30	92	22.28	22.28
	31–40	129	31.23	53.51
	41–50	125	30.27	83.78
	51 and above	67	16.22	100
	Total	413	100	
Education	Primary school	34	8.23	8.23
	High school	70	16.95	25.18
	Intermediate	88	21.31	46.49
	Graduate	144	34.87	81.36
	Postgraduate	77	18.64	100
	Total	413	100	
Monthly income (USD)	Up to 1000	164	39.71	39.71
	1001–2000	113	27.36	67.07
	2001–3000	79	19.13	86.20
	3001 and above	57	13.80	100
	Total	413	100	
Hours spent on online browsing in a week (average)	Up to 5	41	9.93	9.93
	6–10	90	21.79	31.72
	11–15	161	38.98	70.70
	16 and above	121	29.30	100
	Total	413	100	

*USD, United States Dollar.*

Partial least square structural equation modeling (PLS-SEM) was used to analyze the structural associations among the variables of the study (Nunkoo et al., 2013). PLS-SEM was considered appropriate for data analysis for certain reasons, i.e., (1) this study attempts to explain and predict variance in target constructs and sub-constructs, (2) the research paradigm of the present study is complex, (3) the relationships among TDOC, with sub-constructs of OIQ and UFA, satisfaction, and TBI, with sub-constructs of IVTD and eWOM, are new phenomena that endeavor to contribute to the theory of tourism, the internet, online business, and business promotion.

## Results

### Measurement Model Evaluation

The loading values for the endogenous and exogenous study constructs were found to be greater than a 0.70 cut-off point and were significant at the 5% level. The composite reliability of the study constructs was greater than 0.60 and was thus found to be acceptable (Bagozzi and Yi, 1988). The Cronbach’s alpha (α) value was above a 0.70 threshold limit, indicating the reliability of the study constructs (Hair et al., 1998). For the discriminant validity, the study constructs’ average variance extracted (AVE) values were greater than 0.50 and were thus found to be acceptable (Hair et al., 1998), i.e., each AVE square root value was greater than the reflective item’s correlation (Fornell and Larcker, 1981) (see Table 3). Heterotrait-monotrait (HTMT) values (see Table 4) also support the study constructs’ discriminant validity, i.e., less than the 0.85 and 0.90 threshold points (Falk and Miller, 1992).

**TABLE 3:** Measurement model results.

Construct		Item	LV	CR	α	AVE	SQRT(AVE) > Cor^2^	*R*^2^
Tourist destination online content (TDOC)	Online information quality (OIQ)	OIQ1	0.837	0.822	0.797	00.693	0.794 > 0.652	
		OIQ2	0.714					
		OIQ3	0.792					
		OIQ4	0.775					
		OIQ5	0.819					
	User-friendly accessibility (UFA)	UFA1	0.825	0.824	0.786	0.799	0.721 > 0.629	
		UFA2	0.798					
		UFA3	0.781					
		UFA4	0.892					
		UFA5	0.773					
Satisfaction (S)		S1	0.835	0.815	0.771	0.786	0.745 > 0.643	0.733
		S2	0.812					
		S3	0.794					
		S4	0.719					
		S5	0.783					
Tourists’ behavioral intentions (TBI)	Intentions to visit tourist destination (IVTD)	IVTD1	0.829	0.793	0.763	0.718	0.711 > 0.631	0.761
		IVTD2	0.803					
		IVTD3	0.817					
		IVTD4	0.796					
		IVTD5	0.809					
	Electronic word-of-mouth (eWOM)	eWOM1	0.831	0.778	0.808	0.734	0.732 > 0.601	0.815
		eWOM2	0.809					
		eWOM 3	0.782					
		eWOM 4	0.812					
		eWOM 5	0.791					

*LV, loading values; CR, composite reliability; α, Cronbach’s alpha; AVE, average variance extracted; Cor^2^, correlation (highest squared between model constructs); R^2^, co-efficient of determination, level of significance (two-tailed) = 5%.*

**TABLE 4:** Heterotrait monotrait (HTMT) results.

Constructs	*M*	*SD*	OIQ	UFA	S	IVTD	eWOM
Online information quality (OIQ)	3.984	1.517	1.000				
User-friendly accessibility (UFA)	4.205	1.489	0.682^*a*^	1.000			
Satisfaction (S)	4.211	1.483	0.549^*a*^	0.422	1.000		
Intensions to visit tourist destination (IVTD)	3.786	1.691	0.717	0.694	0.591	1.000	
Electronic word-of-mouth (eWOM)	3.773	1.692	0.763	0.773	0.795	0.418	1.000

*^a^Correlation; M = mean; SD = standard deviation.*

### Structural Model Evaluation

**FIGURE 3:**
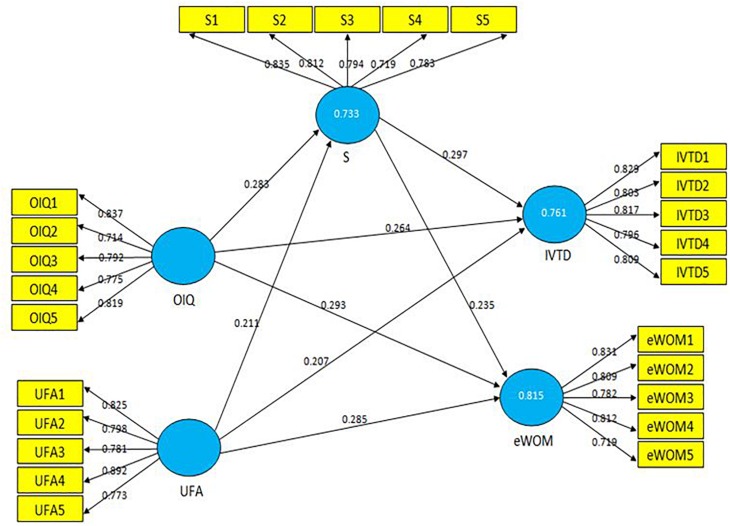
Structural model results.

The coefficient of determination (*r*^2^) values of the mediating and dependent variables were moderately acceptable, i.e., greater than 0.10 (Falk and Miller, 1992), specifically, satisfaction (0.733), IVTD (0.761), and eWOM (0.815) (see Table 3). Structural path coefficients were also calculated. Bootstrapping re-sampling with 5000 replicates was conducted to find *t*-values and standard errors, with each set of the bootstrap sample reporting equal observations to the basic sampling of the present study (Hair et al., 2013), i.e., 413 samples. Table 5 and Figure 3 present the magnitudes and path coefficients of the structural associations among the variables of the study. The effect sizes (*f*^2^ and *q*^2^) of the study items were calculated and found to be acceptable, such as 0.02 (low), 0.15 (moderate), and 0.35 (high) (Chin, 1998) (see Table 5).

**TABLE 5:** Structural model direct effect path coefficients.

Constructs	Standardized coefficients (β)	*t*-values	*f*^2^	*q*^2^	Confidence interval	*p*	Hypothesis confirmation
OIQ → S	0.283	4.147	0.25	0.25	0.197–0.213	0.003	H1 accepted
UFA → S	0.211	3.021	0.19	0.19	0.188–0.259	0.004	
OIQ → IVTD	0.264	3.126	0.21	0.21	0.153–0.204	0.001	H2 accepted
OIQ → eWOM	0.293	4.171	0.19	0.19	0.167–0.211	0.004	
UFA → IVTD	0.207	3.675	0.14	0.14	0.243–0.267	0.001	
UFA → eWOM	0.285	3.299	0.16	0.16	0.206–0.215	0.003	
S → IVTD	0.297	4.352	0.22	0.22	0.155–0.246	0.001	H3 accepted
S → eWOM	0.235	3.663	0.13	0.13	0.164–0.274	0.010	

*OIQ, online information quality; S, satisfaction; UFA, user-friendly accessibility; IVTD, intentions to visit tourist destination; eWOM, electronic word-of-mouth, significance level at 5%.*

The study constructs and their associated significance levels were measured with Beta (β) and *p* values. A significant direct impact of OIQ and UFA on satisfaction was found, i.e., (β = 0.283, *p* = 0.003) and (β = 0.211, *p* = 0.004), respectively, at 5% significance level. Thus, hypothesis H1 is accepted. The findings show significant direct impacts of OIQ on IVTD and eWOM, i.e., (β = 0.264, *p* = 0.001) and (β = 0.293, *p* = 0.004), respectively, at 5% significance level. Likewise, significant direct impacts of UFA on IVTD and eWOM, i.e., (β = 0.207, *p* = 0.001) and (β = 0.285, *p* = 0.003), respectively, at 5% significance level were found, providing support for accepting hypothesis H2. Significant direct impacts of satisfaction on IVTD and eWOM, i.e., (β = 0.297, *p* = 0.001) and (β = 0.235, *p* = 0.010), respectively, at 5% significance level, were found, which provides evidence to accept hypothesis H3 (see Table 5 and Figure 3).

### Mediation Test

A non-parametric bootstrapping approach was adopted, with 5000 bootstrap samples and an equal number of observations as the original sample, to test the mediating impact of satisfaction (Hair et al., 2013). The findings show significant indirect influences of satisfaction on the relationship between OIQ and IVTD (β = 0.191, *t* = 3.023), between OIQ and eWOM (β = 0.247, *t* = 2.169), between UFA and IVTD (β = 0.214, *t* = 2.412), and between UFA and eWOM (β = 0.238, *t* = 2.196). Hypothesis H4 is thus supported. The indirect influence of satisfaction reflects some of the direct impacts of the sub-constructs of TDOC and TBI (Hair et al., 2013). Variance accounted for (VAF) values were calculated to measure the indirect effect size to total size (Hair et al., 2013). The findings show partial mediation among all the constructs of this study (Majeed et al., 2017b) (see Table 6).

**TABLE 6:** Bootstrapping-mediation effects.

Construct	Direct effect-β (*t*-value)	Indirect effect-β (*t*-value)	Total effect	Variance accounted for (VAF)%	Interpretation	Hypothesis
OIQ → S → IVTD	0.264 (3.126)	0.191 (3.023)	0.455	45.19	Partial mediation	H4 accepted
OIQ → S → eWOM	0.293 (4.171)	0.247 (2.169)	0.540	31.23	Partial mediation	
UFA → S → IVTD	0.207 (3.675)	0.214 (2.412)	0.421	66.98	Partial Mediation	
UFA → S → eWOM	0.285 (3.299)	0.238 (2.196)	0.523	77.15	Partial Mediation	

*OIQ, online information quality; S, satisfaction; UFA, user-friendly accessibility; IVTD, intentions to visit tourist destination; eWOM, electronic word-of-mouth; 80% VAF (full mediation), 20–80% VAF (partial mediation); <20% (no mediation) (Majeed et al., 2017b), t — N = 1.96 at p = 0.05 level.*

## Discussion and Implications

Tourists’ favorable behaviors toward a destination’s marketing stimuli hold special relevance for the promotion of tourism business across the globe. The present study is based on an integrated research model that encapsulates tourists’ responses toward TDOC as posted by destinations on the internet and contributes to the theoretical and practical understanding of consumers’ behaviors in the online business environment. This study proposed four hypotheses. The theoretical underpinnings and the proposed hypothesis H1 revealed a significant and positive impact of tourists’ perceptions of TDOC on their satisfaction. The findings show that perceived online information quality has a direct and significantly positive impact on tourists’ satisfaction, i.e., β*_*OIQ*_ → satisfaction* = *0.283*, at *p* < 0.05 where *t* = 4.147. More so, the effect size of the relationship between OIQ and tourists’ satisfaction was 0.25, which is a moderate to high effect size (Chin, 1998; Majeed et al., 2017b). The findings further show that user-friendly accessibility significantly and positively impacted tourists’ satisfaction, i.e., β*_*UFA*_ → satisfaction* = *0.211*, at *p* < 0.05 where *t* = 3.021. The effect size of the relationship between UFA and tourists’ satisfaction was 0.19, which is slightly over moderate effect size (Chin, 1998; Majeed et al., 2017b). Since TDOC is composed of the sub-constructs of online information quality and user-friendly accessibility in this study, the findings of PLS-SEM analysis show a direct and significantly positive impact of tourists’ perceptions of TDOC on their satisfaction. Hypothesis H1 is supported. These findings are partially consistent with the work of Darley et al. (2010) and Sabiote-Ortiz et al. (2016) in terms of the association between consumers’ satisfaction and online information quality.

Hypothesis H2 proposed a significant and positive impact of tourists’ perceptions of TDOC on their behavioral intentions. The findings show 2 × 2 associations of perceptions of TDOC and tourists’ behavioral intentions. First, online information quality has a direct and significantly positive impact on tourists’ intentions to visit tourist destinations, i.e., β*_*OIQ*_ → IVTD* = *0.264*, at *p* < 0.05 where *t* = 3.126 and effect size was 0.21, which was slightly over the moderate level (Majeed et al., 2017b). Second, online information quality has a direct and significantly positive impact on tourists’ eWOM, i.e., β*_*OIQ*_ → eWOM* = *0.293*, at *p* < 0.05 where *t* = 4.171. The effect size of this relationship, i.e., 0.19, was slightly weaker than the effect size of the relationship between OIQ and IVTD but was, however, over the moderate level of 0.15 (Majeed et al., 2017b). Third, the findings show a direct and significantly positive impact of user-friendly accessibility on tourists’ intentions to visit tourist destinations, i.e., β*_*UFA*_ → IVTD* = *0.207*, at *p* < 0.05 where *t* = 3.675. The effect size of this relationship, i.e., 0.14, was marginally lower than the moderate level of 0.15 (Majeed et al., 2017b). Fourth, the findings show a direct and significantly positive impact of user-friendly accessibility on tourists’ eWOM, i.e., β*_*UFA*_ → eWOM* = *0.285*, at *p* < 0.05 where *t* = 3.299, with an effect size over the moderate level, i.e., 0.16 (Majeed et al., 2017b). Since tourists’ behavioral intentions are composed of sub-constructs of tourists’ intentions to visit a tourist destination and eWOM, the 2 × 2 combinations of tourists’ perceptions of TDOC and their behavioral intentions showed significantly positive associations with each other, providing support for hypothesis H2. The effect size of the relationship between user-friendly accessibility and tourists’ intentions to visit a tourist destination was weaker than all of the effect sizes of 2 × 2 combinations of TDOC and tourists’ behavioral intentions. Although the scenarios are different, our findings are in line with the research of Romanazzi et al. (2011) and Chung et al. (2015) in terms of significant relationships being detected between tourists’ perceptions of TDOC and their behavioral intentions.

Hypothesis H3 proposed a significant and positive impact of tourists’ satisfaction on their behavioral intentions. Results show a direct and significantly positive impact of satisfaction on tourists’ intentions to visit a tourist destination, i.e., β*_*satisfaction*_ → IVTD* = *0.297*, at *p* < 0.05 where *t* = 4.352, and eWOM i.e., β*_*satisfaction*_ → eWOM* = *0.235*, at *p* < 0.05 where *t* = 3.663. The effect size of the relationship between satisfaction and eWOM, i.e., 0.13, was weaker than the effect size of the relationship between satisfaction and tourists’ intentions to visit a tourist destination, i.e., 0.22 (Chin, 1998; Majeed et al., 2017b). Hypothesis H3 is supported. More so, hypothesis H4 proposed the mediating impact of satisfaction on tourists’ perceptions of TDOC and behavioral intentions. The 2 × 2 associations of tourists’ perceptions of TDOC and behavioral intentions under the mediating pressure of satisfaction show significantly positive indirect associations, i.e., β*_*OIQ*_ → satisfaction → IVTD* = *0.191* with *t* = 3.023, β*_*OIQ*_ → satisfaction → eWOM* = *0.2.169* with *t* = 2.169, β*_*UFA*_ → satisfaction → IVTD* = *0.214* with *t* = 2.412, and β*_*UFA*_ → satisfaction → eWOM* = *0.238* with *t* = 2.196, at *p* < 0.05. Our findings show that satisfaction exerts its mediating impact on the relationships between the sub-constructs of tourists’ perceptions of TDOC and behavioral intentions, providing support for hypothesis H4. However, PLS-SEM indicates that the mediating impact of satisfaction is partial, considering the VAF values, which are between 0.20 and 0.80 (Majeed et al., 2017b). These findings are partially consistent with the research of Sweeney et al. (2014), which explored associations between tourists’ perceptions, satisfaction, and eWOM.

### Theoretical and Practical Implications

Our findings make important contributions to the tourism and hospitality field. A full mediating influence of tourists’ satisfaction on the relationship between TDOC, with sub-constructs of online information quality and user-friendly accessibility, and tourists’ behavioral intentions, with sub-constructs of tourists’ intentions to visit tourist destination and eWOM, is explored, analyzed, and interpreted. Considering the paucity of empirical research evidence of these interacting phenomena in the online tourism business, our findings from tourists who booked hotels in Lahore, Pakistan, which is a developing country and is a comparatively young market in the Asian tourism and hospitality business, contribute to the fertile grounds of online tourism marketing and destination promotion. Our findings link up the broken connections of tourists’ perceptions and behaviors in the context of TDOC by exploring tourists’ satisfaction as a mediator, which is under-explored in the literature.

The level of tourists’ perceptual risk in online browsing may determine their satisfaction, intentions to visit a tourist destination, and positive or negative eWOM.

Our findings contribute to the research and practice of the tourism and hospitality industry, which endeavor to examine the role of risk and satisfaction in their business functions. High perceived risk in online browsing may negatively impact tourists’ satisfaction and behavioral intentions. Tourist destinations may offer secure online login to satisfy tourists finalizing their online purchase decisions after the initial search for TDOC on different e-platforms. Direct customer service and updated information with easy payment and refund policies at online portals are considered as aspects of online information quality that may help to alleviate the perceptual complexities involved in satisfying tourists in the online tourism business. Strong marketing of tourist destinations and consumer-centered policies may help to satisfy tourists. Such marketing efforts will help to mitigate tourists’ perceptions of risk in online browsing, develop trust (Nunkoo and Ramkissoon, 2012; Nunkoo et al., 2012) between tourists and service providers, and fuel more visits to host tourist destinations and may ensure tourists’ positive eWOM and repeat visits.

Tourists’ information search behaviors in different online channels, such as social media, websites, and e-blogs, have created a plethora of online marketing opportunities for the tourism and hospitality business (Dewnarain et al., 2019). Tourists post their experience of tourism on their social pages, which goes viral with the power of the internet. Tourists’ satisfaction and dissatisfaction with TDOC may determine the success or failure of a tourist destination due to the power of eWOM in the highly competitive online world. The stakeholders of the tourism system need to align their service mechanism in the online environment, which is yet to develop, to serve potential tourists and to remain competitive in the long run. This falls into the dimension of user-friendly accessibility of TDOC. Tourist destinations may offer unrestricted online access to tourists and the option of booking a complete tourism package online, such as tourism and hotel accommodation according to the tourist’s budget, e.g., 2-star to 5-star hotels, guest houses, etc., on their websites or social media pages to offer one-window online scheduling of tourists’ entire itineraries in few clicks.

Stakeholders of tourism services, such as travel agencies, tour operators, and government, consider tourists’ destination selection as an important fuel to their business. During peak tourism periods, tourist destinations receive more tourists, which may gear up the momentum of job creation and have healthy economic impacts on the host country’s economy. Online business promotion costs are negligible as compared to physical marketing and sales promotion efforts. Tourism stakeholders must focus on the notions of online information quality and user-friendly accessibility painted on the canvas of this study under the spotlight of TDOC to strongly promote the online image of their tourist destination and to attract more tourists in both peak times and off-season to gain the maximum benefit for their tourism infrastructure and high profit margins. Thus, tourist destination marketing organizations need to match the pace of the rapidly evolving e-commerce industry to remain competitive in the long run.

## Limitations and Future Research Directions

This study has several limitations that point to directions for future research endeavors. This research was conducted in Lahore, the capital city of Punjab province in Pakistan. Different countries have distinct cultural norms that may influence consumers’ behaviors. Thus, future research may test the findings of this study in different cultures, with culture as a moderator, to extend the scope of TDOC. Our findings are based on data collected by hotel managers without directly contacting tourists. Although such data-collection problems have been encountered previously in hospitality research, future research may attempt to conduct similar studies or validate the findings of the present study by directly contacting tourists staying in hotels. This research is based on a survey research design. Although our findings contribute to the emerging domains of tourism and hospitality in the online environment, future research may incorporate experimental settings to measure the perceptual responses of tourists to online marketing stimuli and TDOC.

## Conclusion

Destinations are encouraged to develop their tourism business along with the online environment to retain existing consumers and attract new tourists. For this, strategic planning is required to maintain the quality of TDOC alongside its user-friendly accessibility. Effective measures to influence the perceptual filters of tourists are needed alongside minimizing the threats to the online tourism business, which ultimately determine tourists’ satisfaction, intentions to visit a tourist destination, and eWOM behaviors. Tourism stakeholders must develop strong coordination (Nunkoo and Ramkissoon, 2016) to provide real-time service to tourists who attempt to book their entire tourism itineraries from their desktops, instilling confidence in quick processing and keeping their information confidential to ensure tourists’ social and economic welfare. This will help to gear-up the momentum of promoting tourism business using the advantages of wireless technology and the internet in parallel to spreading positive eWOM, which is the most trusted tool for attracting customers in these business settings.

## Data Availability Statement

All datasets generated for this study are included in the article/Supplementary Material.

## Ethics Statement

The study was conducted within the ethical limits and by following the protocols approved by the Shenzhen University. Written informed consent was obtained from the study respondents during the data collection process.

## Author Contributions

SM: conceptualization, conduct of the survey, data gathering, data analysis, revisions, development, and proofreading of the article. ZZ and CL: data analysis, revisions, development, and proofreading of the article. HR: revisions, development, and proofreading of the article.

## Conflict of Interest

The authors declare that the research was conducted in the absence of any commercial or financial relationships that could be construed as a potential conflict of interest.
