# Supplementary material for: Online Tourism Information and Tourist Behavior: A Structural Equation Modeling Analysis Based on a Self-Administered Survey
Source: Front Psychol. 2020 Apr 21;11:599. doi: 10.3389/fpsyg.2020.00599 (PMC7186422; doi:10.3389/fpsyg.2020.00599)
Supplement: Supplementary file 1 [file Data_Sheet_1.docx]

**Appendix 1 Questionnaire items**

| No. | Constructs and items | **Agreement** | | | | | | |
| --- | --- | --- | --- | --- | --- | --- | --- | --- |
|  |  | **Strongly disagree Strongly agree** | | | | | | |
|  |  | Strongly disagree | disagree | Somewhat disagree | Neutral | Somewhat agree | Agree | Strongly agree |
|  | **Tourist destination online contents (TDOC)** |  |  |  |  |  |  |  |
|  | **Online information quality (OIQ)** |  |  |  |  |  |  |  |
| 1 | I believe that tourist destination’s online information will be helpful to book the best tourism package. | 1 | 2 | 3 | 4 | 5 | 6 | 7 |
| 2 | I feel that detailed online information about tourist destination will help to satisfy tourists. | 1 | 2 | 3 | 4 | 5 | 6 | 7 |
| 3 | I believe that the updated online price of tourism package will help tourists to quickly decide their tourism schedule. | 1 | 2 | 3 | 4 | 5 | 6 | 7 |
| 4 | I feel that the online quality service will be reliable to meet tourists’ needs and expectations. | 1 | 2 | 3 | 4 | 5 | 6 | 7 |
| 5 | Online view of aesthetic locations of a destination will attract tourists. | 1 | 2 | 3 | 4 | 5 | 6 | 7 |
|  | **User-friendly accessibility (UFA)** |  |  |  |  |  |  |  |
| 6 | I believe that tourist destination’s online content will be easily understandable. | 1 | 2 | 3 | 4 | 5 | 6 | 7 |
| 7 | I believe that online tourism information will help to quickly access the desired information about tourist destination. | 1 | 2 | 3 | 4 | 5 | 6 | 7 |
| 8 | I believe that tourist destination’s online representative will be friendly to tourists. | 1 | 2 | 3 | 4 | 5 | 6 | 7 |
| 9 | I expect that tourist destination’s online information will be easily available on the internet. | 1 | 2 | 3 | 4 | 5 | 6 | 7 |
| 10 | Authentic and updated online tourism information will make tourists relaxed while browsing their desired tourism information. | 1 | 2 | 3 | 4 | 5 | 6 | 7 |
|  | **Satisfaction (S)** |  |  |  |  |  |  |  |
| 11 | Online tourism information delivers positive image of a destination among tourists. | 1 | 2 | 3 | 4 | 5 | 6 | 7 |
| 12 | A destination may present its strong image to tourists with online tourism contents. | 1 | 2 | 3 | 4 | 5 | 6 | 7 |
| 13 | Reliable online tourism information will make tourists happy to purchase online tourism packages. | 1 | 2 | 3 | 4 | 5 | 6 | 7 |
| 14 | Updated online tourism information will make a destination popular among tourists. | 1 | 2 | 3 | 4 | 5 | 6 | 7 |
| 15 | Online tourism information makes tourists relaxed while browsing online tourism destinations. | 1 | 2 | 3 | 4 | 5 | 6 | 7 |
|  | **Tourists’ Behavioral Intentions (TBI)** |  |  |  |  |  |  |  |
|  | **Intentions to visit tourist destination (IVTD)** |  |  |  |  |  |  |  |
| 16 | I will purchase online tourism package to visit my friends and family in host destination. | 1 | 2 | 3 | 4 | 5 | 6 | 7 |
| 17 | Online tourism information will help to easily schedule all fun and adventure activities at host destination. | 1 | 2 | 3 | 4 | 5 | 6 | 7 |
| 18 | Online information about the rest and relaxation activities at host tourism destination will promote tourists’ visits. | 1 | 2 | 3 | 4 | 5 | 6 | 7 |
| 19 | Online tourism promotion with religious facilities will ensure more tourists’ arrivals at host tourism destination. | 1 | 2 | 3 | 4 | 5 | 6 | 7 |
| 20 | Online tourism package will offer greater value of money for my actual traveling to tourism destination. | 1 | 2 | 3 | 4 | 5 | 6 | 7 |
|  | **Electronic word-of-mouth (eWOM)** |  |  |  |  |  |  |  |
| 21 | I will share online comments about my tourism experience. | 1 | 2 | 3 | 4 | 5 | 6 | 7 |
| 22 | I will encourage more tourists online to travel to my experienced tourism destination. | 1 | 2 | 3 | 4 | 5 | 6 | 7 |
| 23 | My shared online comments will generate awareness among tourists about their traveling to the same destination. | 1 | 2 | 3 | 4 | 5 | 6 | 7 |
| 24 | Comments on the positive experience of online tourism booking will help to satisfy tourists about their future intentions to visit the same destination. | 1 | 2 | 3 | 4 | 5 | 6 | 7 |
| 25 | I will communicate my feelings of tourism experience with my friends and family on different online social media. | 1 | 2 | 3 | 4 | 5 | 6 | 7 |
|  |  |  |  |  |  |  |  |  |
